# Experimental models of scald burns. A scope review^1^

**DOI:** 10.1590/s0102-865020190100000007

**Published:** 2019-12-09

**Authors:** Taís Amadio Menegat, Andrea Fernandes de Oliveira, Michelle Gioia Coiado Majewski, Leila Blanes, Yara Juliano, Neil Ferreira Novo, Lydia Masako Ferreira

**Affiliations:** I Fellow Master degree, Postgraduate Program in Translational Surgery, Division of Plastic Surgery, Department of Surgery, Universidade Federal de São Paulo (UNIFESP), Brazil. Conception and design of the study; acquisition, analysis and interpretation of data; technical procedures; manuscript preparation and writing; II PhD, Health Sciences, Postgraduate Program in Translational Surgery, UNIFESP, Sao Paulo-SP, Brazil. Conception and design of the study; acquisition, analysis and interpretation of data; manuscript writing; critical revision; final approval; III Graduate student, School of Medicine, PIBIC/CNPq, UNIFESP, Sao Paulo-SP, Brazil. Conception of the study; IV Full Professor, Biostatistics Department, Universidade Santo Amaro (UNISA), Sao Paulo-SP, Brazil. Technical procedures, interpretation of data, statistical analysis; V Head, Full Professor, Division of Plastic Surgery, UNIFESP. Researcher 1A-CNPq, Director Medicine III-CAPES, Sao Paulo-SP, Brazil. Intellectual, scientific, conception and design of the study; critical revision; CAPES, Sao Paulo, SP, Brazil

**Keywords:** Models, Animal, Animal Experimentation, Mice

## Abstract

**Purpose:**

To conduct a scope review of the experimental model described by Walker and Mason, by identifying and analyzing the details of the method.

**Methods:**

The authors searched Pubmed-Medline, Cochrane-Bireme and PEDro databases for articles published between January 2016 and December 2018, using the following search queries: burns, burn injuries, models animal, and animal experimentation. All articles whose authors used Walker and Mason's model - with or without changes to the method in Wistar rats - were included in this study.

**Results:**

The search identified 45 mentions of Walker and Mason's model; however, after reading each summary, 20 were excluded (of which 5 due to duplicity). The inconsistencies observed after the scope review were: water temperature, length of time of exposure of the experimental model's skin to water, extent of the burnt area, and the description of the thickness/depth of the injury.

**Conclusions:**

Reproducibility of a scientific method is the basis to prove the veracity of the observed results. Thus, it is necessary to have a greater number of publications that adopt a reproducible scientific method, for this review found inconsistencies in the description of Walker and Mason's model.

## Introduction

Superheated liquid is the most frequent among all causes of burns^1^ ^–^ ^3^. Several researches have been conducted with experimental models with hot water burns, thus making it possible to prove and improve methods and procedures, and to allow better understanding of the physiological processes that occur in the injury^4^.

The animal model must be as similar as possible, in functional terms, to the object of the research, as well as it is essential to constantly develop models for each disease or disorder^4^. The model with heated liquid typically employs a mold with an opening through which part of the animal´s body is immersed in boiling water during certain amounts of time, and the depth of the burn is defined by the temperature of the agent, time of exposure and pressure of contact^5^.

The most utilized model used in studies is that of Walker and Mason^6^: a thin asbestos metal cylinder base is cut in half longitudinally, and the extremities are welded to fix two crossed straps of heavy plastic to keep the animal immobilized at the base. The hole of the cylinder is opened and covered with rubber, the model assembly is protected with three layers of adhesive surgical tape and sprayed with a water repellent to avoid leaking of the liquid outside the preset margins. In addition, metal tacks are placed on the four extremities to attach the animal´s paws, as well as clamps to hold the device by its extremity and thus maintain the exposed area immersed in boiling water for 10 seconds (s), resulting in full thickness burn. A 3 s-immersion results in partial burn.

This model, however, and due to the lack of a histological study, does not accurately describe the location of the injury. It only informs if the burn is full thickness or partial. It also does not describe in detail the protocol followed to induce the injury, on account of which some authors that used the same method made changes to it but did not describe them in their studies^7^ ^–^ ^12^. The lack of accuracy and details are the reasons that led us to conduct a scope review, mapping researches made in this field and identifying possible gaps in the model.

## Methods

The study was designed as a scope review in order to map the main concepts that support a specific area of knowledge, to examine the extent, reach, and nature of the investigation, to summarize and publish its data, and to identify the gaps observed in existing studies. Studies that dealt with Walker and Mason´s scalding model in Wistar rats were analyzed, regardless of the object of such studies. The contexts of interest were any in regard to scalding models.

The authors searched Pubmed/Medline, Cochrane/Bireme and PEDro databases for articles in Portuguese, English and/or Spanish, published between 2016 and 2018, that described experiments in animals, using burns, burn injuries, models animal, and animal experimentation as search queries.

The titles and abstracts – if available – of the 45 articles resulting from the search were read and analyzed in order to identify those that were potentially suitable for this study. In case of doubt, they were kept for the next phase, which included reading of the selected articles in full.

The authors included all articles that mentioned having employed Walker and Mason´s model^6^, with or without changes to the method, and excluded those whose objective were not scalding burns and that did not employ Walker and Mason´s method^6^.

Publishing data (for example, year, place and journal) and publication content type (for example, pathology and medication) were extracted. The main focuses of the proposition of the problem, and in the methods, discussions and conclusions were identified, extracted and analyzed. When necessary, the authors would reexamine the full article(s). The authors then identified analysis categories that made it possible to summarize the findings in a narrative manner.

## Results

**Figure 1:**
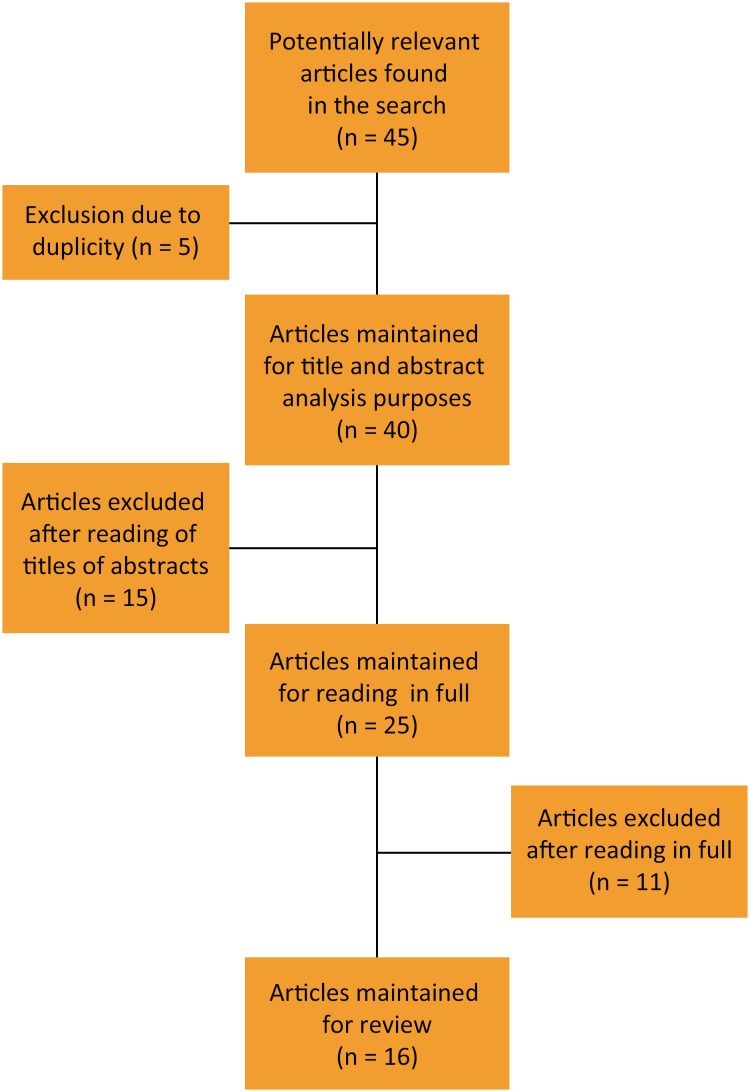
Literature search and inclusion of articles.

The authors’ query-based search strategies produced 45 citations with the model and/or burn descriptor, 5 of which were excluded on account of duplicity. After analyzing the titles and abstracts of the 40 articles pertaining to the query that included the descriptor “scalding”, which is the object of interest of this study, the authors eliminated 15, and subsequently read the remaining 25 articles in full. Finally, 14 articles were chosen and maintained for this review for they were specifically about water scalding using Walker and Mason´s model^6^, with or without changes to the method. Figure 1 describes the analysis flow.

### Objectives of the studies

Among the 16 final remaining articles included in the review for in-depth analysis, 6 (29%) studied some type of burn treatment and 10 (71%) examined some item regarding the pathology itself.

### Temperature and length of exposure time

The sum of the water temperature and the length of exposure time to water is important to determine the injury degree: in 2 studies, temperature ranged from 70°C to 78°C (14%); in 3 studies, from 80°C to 89°C; in 3 other studies, from 90°C to 98°C (21%); in 4 studies, at 100°C (29%); 1 study only mentioned boiling water (7%); and, in 3 studies, the authors do not inform temperature or length of exposure time (21%).

As to length of time of contact with hot water, in 2 studies it is 3 s (14%); in 6 studies, 9 s (6%); in 5 other studies, 10 s (50%); in 1 study, 17 s (7%); and, in 2 other studies, 30 s (14%).

### Injury area

None of the 16 studies describe the exact location, but rather use the words ‘dorsal’ and/or ‘ventral’: 2 studies mention ventral (14%); 13 mention dorsal (85%), and 1 study does not mention the location at all (1%).

As to the injury area, 12 studies used the following formula: TBSA (cm^2^) = 9.1 BW (animal´s body weight in grams) /3; only Walker´s study calculated the area; the other articles did not give any description (86%), and 2 studies measured the area in cm^2^ (14%).

This is an important fact to determine the type of burn. Some studies mention degrees while others mention extent. We observed fourth degree burns in 4 studies (29%), second degree burn in 1 study (7%), and first degree burn in 1 other study (7%). We also observed the terms total/partial thickness in 5 studies (36%), and no mention of the type of burn in 3 studies (21%).

### Description of the burn model

The description of the burn inducing method is important to be able to reproduce the study. Of the studies we reviewed, 6 (43%) inform they followed Walker and Mason´s model ^[^ ^6^ ^]^, but did not inform if they did or not make changes to the model. On the other hand, 8 studies inform having made changes to the model, but do not describe the changes made (57%) (Table 1).

**Table 1:** Description of te burn model.

Author	Objective	Length of Time	Area of Injury	Temperature	Type of burn
Anderson *et al*.^13^	Demonstrate the role of chromium in response to severe burn	–	20%	–	3rd degree burn
Bortolin *et al*.^14^	Burn injury induces histopathological changes in liver of rats	10 s 3 s	30% Dorsal 15% Ventral	87ºC	–
Canhão *et al*.^15^	Effects of mesalamine treatment on gut barrier integrity after burn injury	7 s 9 s	20%	85ºC 95°C	Full thickness 3rd degree burn
Li *et al*.^16^	Auricular vagal nerve stimulation ameliorates burn-induced gastric dysmotility via sympathetic-COX-2 pathways in rats	10 s 3 s	60% Dorsal Ventral	95°C	3rd degree burn
Ida *et al*.^17^	Burn depth assessments by photoacoustic imaging and laser Doppler imaging	10 s	4×10cm^2^ Dorsal	70ºC, 78ºC, 83ºC, 88ºC, 93ºC, 98ºC	Superficial dermal burn, deep dermal burn, and deep burn
Hoscheit *et al*.^18^	Burn injury has skeletal site-specific effects on bone integrity and markers of bone remodeling	30 s	30% Dorsal	100ºC	4th degree burn
Mesquita *et al*.^19^	Effect of low-intensity therapeutic ultrasound on wound healing in rats subjected to third degree burns	10 s	30% Dorsal	100ºC	3rd degree burn
Vana *et al*.^20^	Proteasome inhibition after burn injury	17 s	30-40% dorsal	Boiling water	4th degree burn
Tian *et al*.^21^	The relationship between inflammation and impaired wound healing in a diabetic rat burn model	8 s	10% Dorsal	70ºC	2nd degree burn
Wiggins-Dohlvik *et al*.^22^	Investigate microvascular abnormalities and changes in permeability associated with burn trauma	30% dorsal	10s	100ºC	–
Al-Roujayee^23^	Evaluate the effect of the phenolic compound naringenin on thermal burn-induced inflammatory responses and oxidative stress	10 s	20% Area: 6cm^2^	90ºC	1st degree burns with partial skin injury
Koami *et al*.^24^	Haptoglobin reduces inflammatory cytokine INF-γ and facilitates clot formation in acute severe burn model	–	30% Dorsal	–	4th degree burn
Wiggins-Dohlvik; Tharakan^25^	A rat burn injury model for studying changes in microvascular permeability	10 s	30%	100°C	–
Ida *et al*.^26^	Lensless high-resolution photoacoustic imaging scanner for *in vivo* skin imaging	–	–	–	Deep dermal burn and deep burn

The inconsistencies observed after the scope review were: water temperature, length of time of contact of the experimental model´s skin with water, calculation of the extent of the burnt area and description of the thickness of the injury.

## Discussion

Scalding burns are extremely damaging wounds that disproportionately affect people in developing countries, such as Brazil, where great part of the population lives in unsafe conditions and access to burn treatment is limited. Children up to age 6 are constant victims of scalding/ hot liquids and combustion (chemical) burns, the majority of which are domestic accidents, and represent 60% of the cases. Not only the mortality rate is very high, but also the survivors are burdened with physical and emotional scars for the rest of their lives. The etiology and nature of the injuries caused by scalding burns are different from other causes of burns. Many treatments are controversial and the costs are extremely high. Treatment and management of a scalding burn victim require well trained professionals that are fully aware of the etiology and have access to proper equipment and materials. A burn victim is a challenge for all health professionals, and their continuous improvement in the area is necessary^27^ ^,^ ^28^.

Walker and Mason^6^ created and described the first scalding model, which is still used by researchers, with or without changes. When changes are made to an experimental model, it is extremely important to provide a detailed description of the changes in order to help reproduce the study. In the last years, there has been a small but constant increase in the number of studies/articles about burns, most of which are original articles published in surgical medical journals. These publications were not financially influenced, great part of them was produced in universities, and English is the idiom mostly used to write these articles^27^ ^,^ ^28^.

Walker and Mason^6^ used 100°C as water temperature and 4 studies^18^ ^,^ ^19^ ^,^ ^22^ ^,^ ^25^ did the same. More recent studies^16^ ^,^ ^17^ ^,^ ^23^ used 90°C to 98°C as water temperatures, yet, the results were the same as those using 100°C; 2 studies ^[^ ^17^ ^,^ ^21^ ^]^ used 70°C to 78°C so as to obtain a more superficial injury; and 3 studies^14^ ^,^ ^15^ ^,^ ^17^ used 80°C a 89°C and observed a median injury. The sum of the water temperature and the length of exposure time to the water are important to determine the degree of the injury. Vana *et al*.^20^ only mention ‘boiling water’, which is relative, for the boiling point of water depends on the altitude of the environment. Three studies^13^ ^,^ ^24^ ^,^ ^26^ did not inform the temperature and the length of exposure time to water; therefore, they are not reproducible. As already mentioned above, in order to determine the depth of the injury, it is necessary to associate the water temperature with the length of exposure time of the animal´s skin to it. Walker and Mason ^[^ ^6^ ^]^ used three different lengths of time – 3, 5 and 10 seconds – to obtain three types of injury depth. Two studies^14^ ^,^ ^16^ used 3 seconds and observed a superficial (first degree) burn, which is consistent with the original study. Canhão *et al*.^15^ used 7 to 9 seconds to obtain a median injury (second degree); however, Walker and Mason^6^ observed the same result using 4 seconds. In order to observe a full thickness burn (third degree), 7 studies used the same amount of seconds as Walker and Mason, i.e., 10 seconds^14^ ^,^ ^16^ ^,^ ^17^ ^,^ ^19^ ^,^ ^22^ ^,^ ^23^ ^,^ ^25^. Vana *et al*.^20^ used 16 seconds and Hoscheit *et al*.^18^ 30 seconds. All the authors mentioned having observed deep and severe injury using >10 seconds.

The location and size of the injury depend on the objective of the study, but it is essential to accurately describe the location and the formula used to obtain the size of the injury in order to reproduce the model. No study included in this review described precisely how the location of the injury was obtained as did Walker and Mason^6^, only described as dorsal, without providing details of where on the dorsal area^14^ ^,^ ^16^ ^–^ ^22^ ^,^ ^24^. Bortolin *et al*.^14^ and Li *et al*.^16^ informed the location as ventral, but without further details in order to allow reproducibility.

All aspects of a study must be fully described in order to make it reproducible. We did find two circumstances in studies that followed Walker and Mason´s model^6^; however, the authors did not inform if they made changes to the model^13^ ^,^ ^14^ ^,^ ^19^ ^,^ ^20^ ^,^ ^23^. In reading the articles in full, we noticed changes in temperature, location and injury extent. We also read studies that confirm having made changes to the model, but do not mention what kind of changes were made^15^ ^–^ ^18^ ^,^ ^21^ ^,^ ^24^ ^–^ ^26^, thus making it impossible to reproduce the model.

## Conclusions

Reproducibility of a scientific method is the basis to prove the veracity of the observed results. Thus, it is necessary to have a greater number of publications that adopt a reproducible scientific method, for this review found inconsistencies in the description of Walker and Mason´s model.
